# Phenytoin- Medication That Warrants Deviation From Standard Approach for Thyroid Lab Interpretation

**DOI:** 10.7759/cureus.11324

**Published:** 2020-11-04

**Authors:** Vishwanath Pattan, Narsimha Candula, Ramesh Adhikari, Rahul Kashyap

**Affiliations:** 1 Endocrinology, Wyoming Medical Center, Casper, USA; 2 Hospital Medicine, University of Florida Health Jacksonville, Jacksonville, USA; 3 Hospital Medicine, Franciscan Health, Lafayette, USA; 4 Department of Anesthesiology and Peri-operative Medicine, Mayo Clinic, Rochester, USA

**Keywords:** central hypothyroidism, phenytoin, lab interpretation, low free t4

## Abstract

Long-term treatment with antiepileptic drugs like phenytoin has been reported to alter the thyroid hormone levels. It makes interpretation of thyroid labs hard for clinicians. We report a case of 48-year-old Caucasian female on phenytoin since year 1996 with stable seizure control, who was referred to endocrinology clinic in 2016 for evaluation of suspected central hypothyroidism due to discordant results in thyroid lab panel. Labs showed decreased free T4 level of 0.68ng/dL (reference range 0.89-1.76ng/dL) in the setting of normal thyroid stimulating hormone (TSH) 1.76 µIU/mL (reference range 0.46-4.68 µIU/mL). Clinically patient was euthyroid. Free T3 level was normal -3.82 pg/mL (reference range 2.77-5.27 pg/mL). Phenytoin was identified as the cause of the artifactual lowering of free T4 on routine assays. Therefore subsequent thyroid monitoring was done with TSH measurements. Continued follow-up of TSH remained normal over the subsequent follow-up of four years.

## Introduction

Epilepsy is a common neurological disorder that requires most patients to remain on long-term anti-epileptic drugs. Both conventional and newer anti-epileptic medications have been reported to significantly alter thyroid hormone levels [1]. Thyroid stimulating hormone (TSH) and free T4 levels are commonly ordered thyroid labs to assess thyroid function. However interpretation of these labs can be confusing to clinicians in some patients who are on phenytoin. In this case report we review the changes caused by phenytoin on thyroid hormone metabolism which influences thyroid lab interpretation and hence management.

## Case presentation

A 48-year-old post-menopausal Caucasian female was referred to the endocrinology clinic in September 2016 because of suspected central hypothyroidism. Past medical history was significant for epilepsy, osteopenia, and vitamin D deficiency. Patient presented to her primary care physician with symptoms of fatigue. She had screening for sleep apnea and iron deficiency which were normal. Patient had thyroid labs checked on July 29, 2015, which showed TSH 1.06 µIU/mL (reference range 0.46-4.6 µIU/mL), free T4 0.83 ng/dL (reference range 0.89-1.76 ng/dL). Other than mild fatigue, patient was asymptomatic. Thyroid labs on February 23, 2016, showed TSH 1.67 µIU/mL, free T4 0.85 ng/dL. Because of persistently low free T4, a repeat thyroid lab panel was done again on August 15, 2016, which showed a further drop in free T4 to 0.68 ng/dL and TSH remained normal at 1.76 µIU/mL. Because of the significant drop in free T4 in the setting of normal TSH, central hypothyroidism was suspected and patient was referred to endocrinology.

On examination, the thyroid gland was barely palpable without palpable nodules. Visual field examination by finger confrontation was normal. Patient did not use biotin and there was no prior use of iodinated contrast before the thyroid labs. Review of the thyroid labs (all available labs summarized in Table 1) showed persistently suppressed free T4 values but normal values for TSH. Repeat thyroid panel on September 8, 2016 showed TSH 1.96, free T4 0.68 ng/dL, total T4 -4.2 µg/dL (reference range 4.5-11.7 µg/dL), total T3 1.18 ng/mL (reference range 0.97-1.69 ng/mL), free T3 3.82 pg/mL(reference range 2.77-5.27 pg/mL). Thyroid peroxidase antibody was 0.3 IU/mL (reference range 0-9 IU/mL). Total T3 and free T3 levels were normal but free T4 and total T4 levels were low. Thyroid ultrasound done six months prior showed normal size of thyroid gland without any discrete nodules. Hormonal evaluations done to evaluate her symptoms of fatigue were normal. Adrenocorticotropic hormone (ACTH) stimulation test showed baseline ACTH 13 pg/mL (reference range 10-60 pg/mL), baseline cortisol 8.6 mcg/dL, 60 minute cortisol 20.4 mcg/dL, dehydroepiandrosterone sulfate 32.5 mcg/dL (reference range 18-244 mcg/dL), follicle-stimulating hormone (FSH) 71.5 IU/L (postmenopausal reference range at 16.7-113.6 IU/L), insulin-like growth factor-1 134 ng/mL (reference range 44-227 ng/mL), prolactin 7.1 ng/mL (4.8-23.3 ng/mL).

**Table 1:** Serial monitoring of thyroid labs and Phenytoin concentration T3- Triiodothyronine T4- Tetraiodothyronine TSH- Thyroid stimulating hormone Anti-TPO antibodies: Anti-Thyroperoxidase antibodies (L): lab value is below normal range

Lab values (reference range) & Date	Free T3 (2.77-5.27 pg/mL)	Total T3 (0.97-1.69 ng/mL)	Free T4 (0.89-1.76 ng/dL)	Total T4 (4.5-11.7 µg/dL)	TSH (0.46-4.68 µIU/mL)	Anti-TPO antibodies (0-9 IU/mL)	Phenytoin concentration (10-20 µg/mL)
7/29/2015			0.83 (L)		1.06		
2/23/2016			0.85 (L)		1.67		
8/15/2016			0.68 (L)		1.76		
9/8/2016	3.82	1.18	0.68 (L)	4.2 (L)	1.96	0.3	9
10/24/2016		1.04	0.77 (L)		1.87		
3/30/2017					1.5		
1/19/2018					2.36		7
8/20/2019					2.27		6

Review of home medications revealed that patient had been taking phenytoin 300mg daily since 1996 with stable seizure control. Because of suppressed free T4 concentration, central hypothyroidism was suspected by the primary care physician. However, the use of phenytoin is reported to mimic central hypothyroidism. Although phenytoin is known to displace T4 from binding proteins that could cause a rise in free T4 and reciprocal fall in TSH, both free T4 and TSH normalize after equilibrium is reached. Decreased free T4 levels observed in serum of phenytoin-treated patients on routine lab assays have been shown to be artifactual. Therefore the decision was made to rely on TSH measurements to confirm the euthyroid status as long as patient remained on phenytoin. Fatigue symptoms resolved in the coming months and no clear etiology of fatigue was found. Subsequent measurements of TSH remained normal over the next four years while patient stayed on phenytoin.

## Discussion

Most thyroid function lab panels are straightforward to interpret. However in certain subgroups of patients, the labs may appear incongruent with each other, and hence discordant with the clinical picture. Patients with epilepsy often remain on antiepileptic drugs for a long period of time. Several antiepileptic medications like phenytoin and carbamazepine are known to influence serum concentrations of thyroid hormones. In our patient, TSH was normal but free T4 levels were consistently suppressed giving a picture of central hypothyroidism to the primary care physician.

Central hypothyroidism is characterized by a defect in thyroid hormone secretion in an otherwise normal thyroid gland due to insufficient stimulation by TSH (due to abnormal function of the pituitary gland and/or hypothalamus). The diagnosis of central hypothyroidism is based on low circulating levels of free T4 in the presence of low to normal TSH concentrations [2].

In such situations it is important to consider the clinical context, potential confounding factors, concurrent medication use, intercurrent illness and other factors that can influence the thyroid function panel in the labs. Our patient was not on biotin, did not have any recent use of iodinated contrast, and did not have any recent acute illness that could influence interpretation of thyroid labs. She had no history of malignancy, head trauma, or radiation exposure. There was no evidence of visual field defect on clinical exam, no biochemical evidence of gonadotropin deficiency, growth hormone (GH) deficiency, or hyperprolactinemia to suggest a central cause. Most patients with a central cause- like pituitary adenoma or mass, radiation and trauma- usually present with a typical course starting with GH (or insulin-like growth factor 1 (IGF-1)) deficiency, gonadotropin deficiency, and then progressing to TSH or ACTH deficiency [3]. Moreover she was using a medication (phenytoin) that is known to cause specific changes in thyroid hormone levels, especially a drop in measured free T4 levels on routine labs (discussed below).

Oppenheimer et al. (1961) studied mean serum protein-bound iodine (PBI) level in 36 phenytoin-treated patients with seizures and reported that phenytoin depresses the level of serum PBI by interfering with the binding of thyroxine by plasma proteins [4]. Based on this finding, one would expect free T4 concentrations to rise. However, the measured free T4 concentrations have been reported to be low in phenytoin-treated patients [5,6], which is also the case in our patient.

This paradox was resolved by Surks et al. (1996) who studied 19 patients on phenytoin with plasma levels in the upper half of the therapeutic range [7]. In vitro, therapeutic concentration of phenytoin resulted in significant increase in free T4 (25% to 88% increase) fraction and a comparable increase in serum-free T4 concentration when measured by ultrafiltration assay. However this increase was not seen when serum free T4 was measured by chemiluminescence assay with 1:5 dilution of serum. In diluted serum, the free drug concentration of phenytoin falls rapidly to a level at which T4 is not significantly displaced from the T4 binding globulin. Thus reduced free T4 levels observed on most routine free T4 assays are artifactual and related to serum dilution used in lab assays [8]. Clinicians should therefore rely on serum TSH concentration rather than serum-free T4 concentration to assess the functional state of thyroid in patients treated with phenytoin.

## Conclusions

Patients treated with phenytoin can have thyroid labs that may mimic central hypothyroidism. Clinicians should be wary of decreased free T4 levels observed on routine lab assays in the serum of patients treated with phenytoin, as this may be artifactual.

Clinicians should rely on TSH to assess thyroid functional status in patients treated with phenytoin in the absence of potential central cause.

## References

[1] Adhimoolam M, Arulmozhi R (2016). Effect of antiepileptic drug therapy on thyroid hormones among adult epileptic patients: an analytical cross-sectional study. J Res Pharm Pract.

[2] Beck-Peccoz P, Rodari G, Giavoli C, Lania A (2017). Central hypothyroidism - a neglected thyroid disorder. Nat Rev Endocrinol.

[3] Kim SY (2015). Diagnosis and treatment of hypopituitarism. Endocrinol Metab (Seoul).

[4] Oppenheimer JH, Fisher LV, Nelson KM, Jailer JW (1961). Depression of the serum protein-bound iodine level by diphenylhydantoin. J Clin Endocrinol Metab.

[5] Chin W, Schussler GC (1968). Decreased serum free thyroxine concentration in patients treated with diphenylhydantoin. J Clin Endocrinol.

[6] Larsen PR, Atkinson AJ Jr, Wellman HN, Goldsmith RE (1970). The effect of diphenylhydantoin on thyroxine metabolism in man. J Clin Invest.

[7] Surks MI, DeFesi CR (1996). Normal serum free thyroid hormone concentrations in patients treated with phenytoin or carbamazepine. A paradox resolved. JAMA.

[8] Burch HB (2019). Drug effects on the thyroid. N Engl J Med.

